# Leiomyomatosis Peritonealis Disseminata with Sarcomatous Transformation: A Rare Case Report and Literature Review

**DOI:** 10.1155/2019/3684282

**Published:** 2019-12-05

**Authors:** Song Xu, Jing Qian

**Affiliations:** Department of Gynaecology and Obstetrics, Hangzhou First People's Hospital, Zhejiang University School of Medicine, No. 261 Huansha Road, Hangzhou, Zhejiang, China

## Abstract

Leiomyomatosis Peritonealis Disseminata (LPD) is an uncommon disease characterized by presence of Multiple Leiomyomas in the abdominal and pelvic cavity. Increasingly, literature supports that LPD is an iatrogenic condition. Malignant transformation of LPD is rarely reported. We hereby report a case of a middle-aged female patient who was diagnosed with malignant sarcomatous degeneration in LPD. Gynecologists may get inspiration from this case report.

## 1. Introduction

Leiomyomatosis Peritonealis Disseminata (LPD) is a disease with low incidence which is featured by multiple nodules in the abdominal and pelvic cavity. Malignant transformation of LPD is extremely rare. Here we report a middle-aged female patient who was diagnosed with LPD and malignant sarcomatous degeneration. Recently, increasingly literatures have supported that LPD is an iatrogenic condition due to the use of morcellation. As such we introduce an innovative method to solve this problem which can safely remove the large fibroids in laparoscopic myomectomy without the use of morcellation.

## 2. Case Report

**Figure 1:**
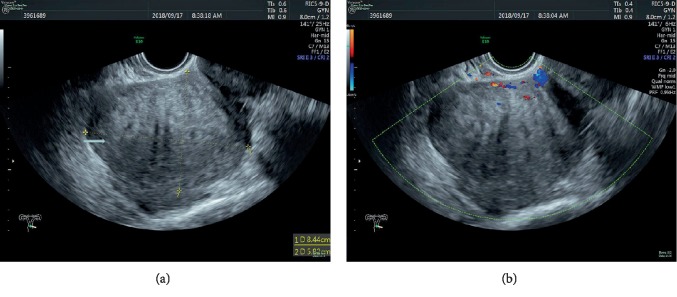
(a) Hypoechoic mass lesion on ultrasound image. (b) Strip-like blood flow signal of the mass on color Doppler flow image.

**Figure 2:**
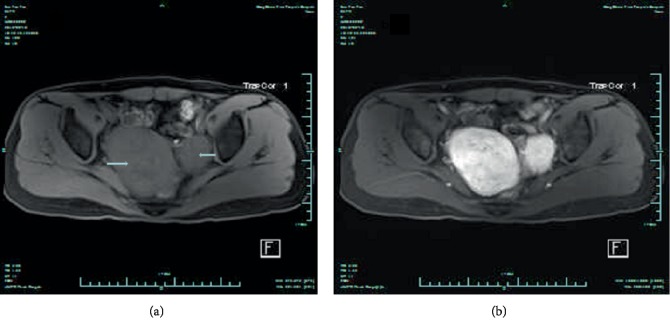
(a) Multiple lobulated, intermediate intensity lesions in T1W image. (b) Intense enhancement of the lesions following contrast administration in T1W image.

A 47-year-old female, with a history of laparoscopic subtotal hysterectomy and bilateral salpingectomy ten years ago, was hospitalized in September 2018. The main reason of the surgery ten years ago was mutiple myomas and the corpus uteri was taken out after the use of morcellation. She presented a rapidly growing tumor in her pelvic cavity, which was about 9 centimeters in diameter when she was hospitalized. The woman's condition was evaluated with transvaginal three-dimensional sonography and magnetic resonance imaging (MRI) after admission. The ultrasound images depicted multiple, solid, hypoechoic mass lesions in pelvis (Figure 1(a)). Color Doppler flow imaging (CDFI) showed strip-like blood flow signal (Figure 1(b)). MRI scans revealed multiple lobulated, intermediate intensity lesions in T1W images (Figure 2(a)). These lesions showed intense enhancement on post enhancement images (Figure 2(b)).

**Figure 3:**
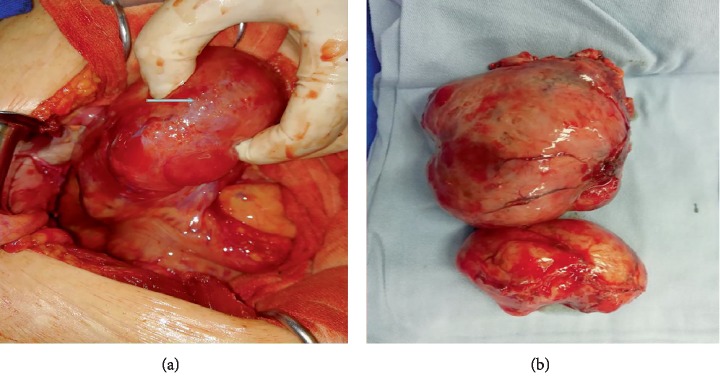
(a) The largest mass on the surface of retroperitoneum. (b) The gross appearance of the larger two lesions.

The patient underwent exploratory laparotomy with bilateral oophorectomy; residual cervix was also removed. All of the mass lesions varying in size from 1 cm to 9.5 cm were resected from the pelvis. These nodules scattered located on the surface of retroperitoneum, sigmoid colon and urinary bladder (Figure 3(a)). All the visible lesions were excised including two biggest nodules on retroperitoneum and sigmoid colon (Figure 3(b)).

**Figure 4:**
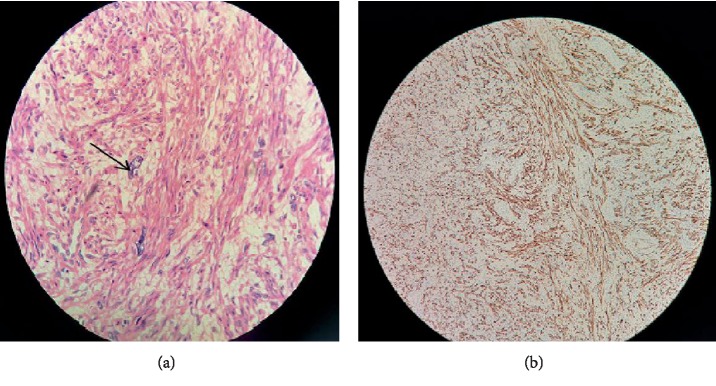
(a) HE staining of the sample under microscope (magnification: 200x). The arrow refers to atypical spindle cells. (b) The positive result of immunohistochemistry of desmin, which is a specific protein of muscle cells.

Intraoperative frozen section revealed spindle-shaped tumor cells with histopathological atypia, which needed routine histologic examination and immunohistochemistry for diagnoses. The operation was successfully performed. Histopathological examination, after the surgery, confirmed the diagnosis of LPD with cellular leiomyoma and sarcoma degeneration. The pelvic nodules were composed of close-arranged spindle cells which were featured by nuclear enlargement, pleomorphism and atypia (Figure 1(a)). On immunohistochemistry, the cells are positive for specific smooth muscle proteins: Desmin[+] (Figure 4(b)), SMA[+], Caldesmon[+]. Proliferation index (Ki-67) was about 5–10%. The patient was given 6 cycles (per 3-4 weeks) of adjuvant chemotherapy of epirubicin (40 mg/m^2^). Three months after the chemotherapy, she remained asymptomatic and no abnormal imaging results were found.

## 3. Discussion

LPD was first reported in 1952 by Wilson [1] but named and described by Taubert in 1965 [2]. About 200 cases of LPD were reported in the English literatures up to date. LPD is a rare condition featured by formation of multiple nodules in the abdominal and pelvic cavity. This condition typically has a similar presentation to malignancy, but its pathological appearance is benign just like uterine myoma. Although LPD is a benign disease, rare cases of malignant transformation have been reported in the literatures [3, 4]. Several theories have been proposed as to the pathogenesis of this disease: hormonal dysfunction, differentiation of subperitoneal mesenchymal stem cells, myofibroblastic metaplasia, genetic and iatrogenic causes (resection of myomas during laparoscopic surgery) [5]. To find this disease is challenging because most of the patients are asymptomatic. Some of them may even be misdiagnosed or never diagnosed for they do not have typical symptoms such as abdominal pain or discomfort, vaginal bleeding abdominal distension, and so on. Imaging examinations including ultrasound (US), computed tomography (CT) and magnetic resonance imaging (MRI) were suggested to evaluate this disease [3, 6]. Definitive diagnosis of LPD should be made by a histopathological examination after laparotomy or laparoscopy. The histologic features of LPD are similar to benign spindle cell tumor. They show smooth-muscle cell proliferation with no atypia or necrosis. Accidentally malignant change may happen just like our case. There is no consensus on the treatment of LPD. Treatments with GnRH agonists [7], aromatase inhibitors [8] were described as effective methods in initial treatment and adjuvant therapy after conservative operation. In case of women with no reproductive desire or post-menopausal women, surgical approach with hysterectomy, salpingo-oophorectomy and debulking surgery was advised. While in case of women with reproductive desire, a conservative approach was preferred [6].

**Figure 5:**
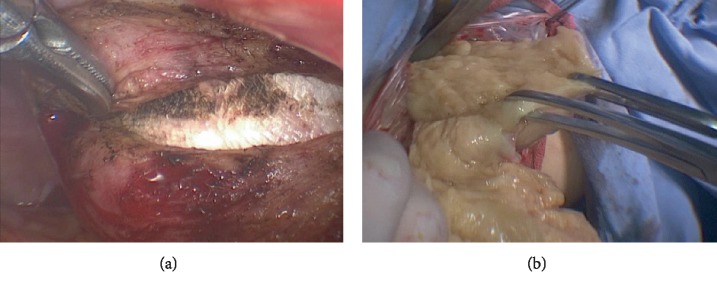
(a) An incision is made in the posterior fornix of vagina. (b) The lesion is placed in a fetch bag and the edge of the bag was pulled out through the incision.

Nowadays increasingly reports described laparoscopic myomectomy and morcellation as the contributing factor of LPD [9, 10]. Morcellation is the fragmentation of tissue to remove specimen through small incisions in minimally invasive surgery. This technique may induce intra-abdominal dispersion of myoma debris, that lead to peritoneal dissemination and worse outcomes. It is possible that the minced fragments of myoma could survive and grow to become LPD. As in our case, LPD with sarcomatous transformation was presented ten years after laparoscopic subtotal hysterectomy. In our opinion, we emphasize the principle of tumor-free technique in laparoscopy. Based on this point of view, stopping the use of morcellation and removing the leiomyoma on large mass rather than fragments would contribute to a decline in the rate of LPD. In our hospital, we use a novel method to retrieve myoma without morcellation in laparoscopy. When the operation is conducted, we make an incision in the posterior fornix of vagina (Figure 5(a)) and put the tumor into a fetch bag, then we pull out the edge of the opening of the bag through vaginal incision and implement manual rotary cut of the lesion to get it out with 100 percent no residue left in the abdomen (Figure 5(b)).

In addition to this most important advantage, the new technique can also shorten operation time, reduce the operation cost and avoid the extension of abdominal incision to remove the tumor. In our experience, this method to remove myoma is easy to grasp and cost-effective. Further randomized controlled clinical trial is needed to evaluate the significance of this method in decreasing the rate of LPD.
